# Manipulating the Cathodic Modification Effect on Corrosion Resistance of High Corrosion-Resistant Titanium Alloy

**DOI:** 10.3390/ma16186217

**Published:** 2023-09-15

**Authors:** Bosung Seo, Hyung-Ki Park, Chang-Soo Park, Seongtak Kim, Kwangsuk Park

**Affiliations:** Gangwon Regional Division, Korea Institute of Industrial Technology, Gangneung-si 25440, Gangwon-do, Republic of Korea; bs3863@kitech.re.kr (B.S.); mse03@kitech.re.kr (H.-K.P.); zionzia@kitech.re.kr (C.-S.P.); seongtak@kitech.re.kr (S.K.)

**Keywords:** corrosion-resistant titanium alloys, tantalum, tantalum oxide, precipitate, corrosion

## Abstract

Further improving the corrosion resistance of the ASTM Grade 13 (Gr13) titanium alloy was achieved by manipulating the cathodic modification effect. The cathodic modification of Gr13 was mainly related to the Ti_2_Ni precipitate, where minor Ru was contained and controlled the precipitate in terms of size and distribution, which could manipulate the cathodic modification effect. Parameters such as temperature and cooling rate during the recrystallization process were designed to control precipitation behavior, where the temperature at 850 °C was selected to allow the full dissolution of the Ti_2_Ni precipitate. The cooling rate, as high as 160.9 °C/min, was still enough for precipitation to occur during the cooling stage, leading to the formation of the Ti_2_Ni precipitate along with a grain boundary. The cooling rate of water quenching was too fast to cause the diffusion process, resulting in a large amount of the β-Ti phase without the precipitate, which was pre-formed while heated at 850 °C. Aging at 600 °C caused the re-precipitation of Ti_2_Ni, and, at that moment, the precipitate was refined and separated, as a good aspect of the catalyst for HER. Therefore, the aged sample after water quenching showed the lowest onset potential for HER with the highest corrosion potential, indicating that its passivation ability was improved by the strengthened cathodic modification effect. This improvement was confirmed by the OCP results, where passivation survival was observed for the aged sample due to the highest cathodic modification effect. Therefore, the aged sample, which had refined and separate precipitates, showed the lowest corrosion rate.

## 1. Introduction

Corrosion is a type of phenomenon of metal deterioration as a result of physicochemical interactions between metals and the environment. In particular, electrochemical corrosion consists of two partial electrochemical reactions, including an anodic reaction (corrosion) and a cathodic reaction, which requires three components, namely anode, cathode and electrolyte. These components should co-exist and contact each other for corrosion occurrence; therefore, if one of them is eliminated or separated, corrosion can be prevented [1,2,3]. Titanium and titanium alloys usually exhibit superior corrosion resistance, even under severe corrosion conditions, such as oxidizing acids (NHO_3_, H_2_CrO_4_), mild reducing acids (most organic acids) and seawater, due to the presence of an ultrathin TiO_2_ film [4,5,6,7]. The inert and self-regenerated characteristics of the TiO_2_ film formed on the titanium surface provide resistance in their reaction with the contacted environment, placing titanium and titanium alloys under separate conditions from the environment. Thus, the performance of the TiO_2_ film in terms of its formation, stability and regeneration ability is crucial when determining the corrosion properties of titanium and titanium alloys.

An approach to improve corrosion resistance by adding elements, there are four possible ways: an increase in thermodynamic stability, the retardation of cathodic reaction kinetics, the formation of a stable passivation layer and the retardation of anodic reaction kinetics [8]. The latest one is usually realized by adding platinum group metals (PGMs) into passivation materials, such as stainless steels and titanium, and is called cathodic modification, where an introduced active site reduces the overpotential of a cathodic reaction, elevating the cathodic reaction in a passivation region [9,10,11,12,13]. The ASTM Grade 13 (Gr13) titanium alloy (Ti-0.5Ni-0.05Ru) is a PGMs-containing titanium alloy, and it also shows the cathodic modification effect due to the presence of the Ti_2_Ni_(1−x)_Ru_x_ precipitate [14,15,16,17]. Ruthenium is known to act as a catalyst with a secondary phase, like a precipitate. Therefore, as a catalyst for hydrogen evolution reaction (HER) on titanium’s surface, the controlled precipitate in terms of size and distribution can alter the corrosion properties of titanium alloys via manipulating cathodic modification [18]. This catalytic effect on HER has often been observed in other works [19,20,21].

The Gr13 titanium alloy is mainly composed of the α-Ti phase, in which the β-Ti phase and Ti_2_Ni precipitate co-exist along with the grain boundary. Because alloying elements, such as Ni and Ru, are β stabilizing elements, a limited amount of the β-Ti phase also remains at room temperature [22,23]. With thermomechanical processes, such as hot rolling and heat treatments, recrystallization heat treatment is also conducted, usually below the β transus temperature ranging from 600 °C to 750 °C, as a final process. Previous results have shown that the Ti_2_Ni precipitate in Gr13 is dissolved at 760 °C, which is a similar temperature to recrystallization, indicating that the Ti_2_Ni precipitate could be dissolved in the α-Ti phase if the recrystallization temperature is higher than 760 °C [24,25]. However, there are few works that consider the morphologies of the Ti_2_Ni precipitate, especially from the viewpoint of cathodic modification. As the final Ti_2_Ni precipitate is the result of reprecipitation when the temperature is high enough to cause the Ti_2_Ni precipitate to dissolve during the recrystallization process, the morphology of the Ti_2_Ni precipitate in terms of size and distribution can be manipulated during the cooling stage, here, precipitation behavior from the solid solution state is controlled by the cooling rate. That is, the final morphology of the precipitate is kinetically determined by a competition between nucleation and growth, and thus, the cooling rate plays a great role in determining the size and distribution of the precipitate. As a catalyst, the refined precipitate exerts a significant impact on HER, thus strengthening the cathodic modification effect.

In this study, different cooling rates were applied to change the morphology of the Ti_2_Ni precipitate in terms of size and distribution. As a catalyst for HER, the Ti_2_Ni_(1−x)_Ru_x_ precipitate plays a critical role in obtaining the cathodic modification effect, and thus, the degree of the cathodic modification effect is enhanced by controlling the size and distribution of the precipitate. Therefore, it is expected that the further enhanced corrosion resistance of Gr13 can be achieved simply by adjusting the processing parameters.

## 2. Materials and Methods

### 2.1. Specimens Preparation

**Figure 1:**
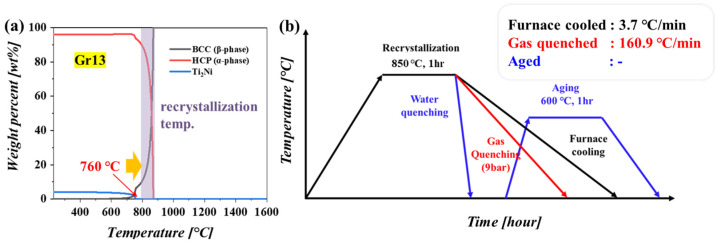
(**a**) Calculated phase diagram of Gr13 and (**b**) Temperature profile for recrystallization process, where the cooling rate was controlled to manipulate precipitation behavior.

The ASTM Grade 13 titanium alloy was used in this study (Ti-0.5Ni-0.05Ru) and fabricated by vacuum arc melting. The alloy ingot was homogenized with a solid solution heat treatment at 1000 °C for 10 h under vacuum conditions. Hot rolling was conducted to break the solidified microstructure, where a total of eight passes (20% reduction per pass) were applied to achieve an 80% reduction in thickness, leading to a final 4 mm thickness. The recrystallization process was conducted at 850 °C under vacuum conditions for the full dissolution of the precipitate (Ti_2_Ni), and then different cooling rates, such as furnace cooling, gas quenching (Argon, 9 bar) and water quenching, were applied to control precipitation behavior. Due to the fact that no precipitate was formed, aging at 600 °C was applied to the water-quenched sample to perform reprecipitation. The phase diagram calculated by the Thermo-Calc program was used to determine the recrystallization temperature, which was higher than the dissolution temperature of the Ti_2_Ni precipitate (760 °C), allowing the full dissolution of the Ti_2_Ni precipitate and temperature profile, especially for the cooling rate during the recrystallization process; this is schematically drawn, as shown in Figure 1. The shadow area in Figure 1, indicating the recrystallization temperature region, was determined by considering the dissolution temperature of the Ti_2_Ni precipitate.

### 2.2. Characterizations

Microstructural observations, depending on the cooling rate, were focused on precipitates in terms of their size, morphology and composition. Scanning electron microscopy (SEM, FEI company, Quanta 250, Hillsboro, OR, USA) and a transmission electron microscope (TEM, JEOL, JEM-2200FS, Tokyo, Japan) were used for microstructural analysis, especially for grain boundaries where the β-Ti phase, as well as Ti_2_Ni precipitates, were formed. The etchant used to reveal the microstructure was deionized water containing 3 wt% HNO_3_, 2 wt% HCl and 2 wt% HF acids. Electrochemical tests (Autolab PGSTAT302N, Metrohm autolab, Utrecht, The Netherlands) such as linear sweep voltammetry, potentiodynamic polarization and open circuit potential (OCP) tests were conducted to characterize the corrosion properties, where the three-electrode configuration with the Pt plate counter electrode and Ag/AgCl reference electrode were used, respectively. Samples for electrochemical tests were prepared by grinding with silicon carbide papers (#800, #1200 and #2400), followed by polishing with 3 µm and 1 µm of diamond paste. The polished surface was then cleaned ultrasonically in an ethanol bath and dried with pressurized air before the electrochemical tests. In total, 10 wt% and 30 wt% of the sulfuric acid (H_2_SO_4_) solution was used as the electrolyte, and nitrogen purge for 30 min was applied to remove dissolved oxygen from the electrolyte before the electrochemical tests. Linear sweep voltammetry was conducted with a scan rate of 0.05 mV/s in a range from 0 to −0.5 V, and potentiodynamic polarization tests (ASTM G5, G61) were performed with a scan rate of 0.05 mV/s in a range from −1.5 V to 1.5 V. Immersion tests were conducted with 30 wt% of the sulfuric acid solution to obtain a corrosion rate based on weight loss. The corrosion rate was calculated according to the ASTM G 31 standard, where weight loss during 24 h was measured to obtain the corrosion rates as follows: [26,27,28,29].

Corrosion rate (mm/y) = K∙W/A∙T∙D(1)

where K is the constant (8.76 × 104), W is the weight loss during 24 h, A is the surface area of a sample, T is time (h) and D is the density of a sample.

## 3. Results and Discussion

### 3.1. Microstructural Characterization with Cooling Rate

**Figure 2:**
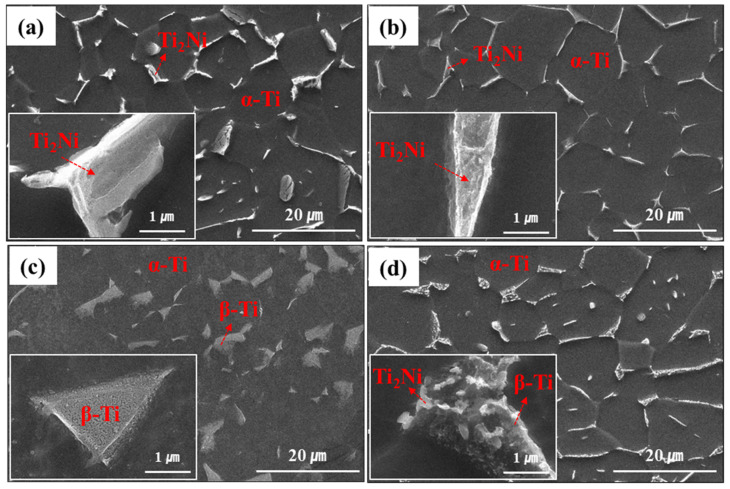
SEM images showing microstructural changes with different cooling conditions: (**a**) Furnace cooling, (**b**) Gas quenching, (**c**) Water quenching and (**d**) Aging at 600 °C after water quenching. The insets in each image highlight the second phases such as Ti_2_Ni precipitate and β-Ti phase.

Due to the addition of alloying elements, such as Ru and Ni, which were β stabilizing elements, the ASTM Grade 13 titanium alloy contained the β-Ti phase as well as the Ti_2_Ni precipitate in the majority of the α-Ti phase, as shown in Figure 2. As reported in previous results, the β-Ti phase and Ti_2_Ni precipitate co-exist along with and/or around the grain boundary, making them appear as one precipitate in the SEM image [24].

The slow cooling rate seemed to cause the growth of the precipitate. Furnace cooling showed a broader precipitate compared to gas quenching, indicating that a slow cooling rate could have a greater chance of growing the precipitate. However, a difference in precipitate size was not obvious between these two cases in spite of a significant difference in the cooling rates (3.7 °C/min vs. 160.9 °C/min). The precipitate became slightly narrower with gas quenching. On the other hand, the fastest cooling rate changed the situation. There was no precipitate formation and transformation (β-Ti → α-Ti) during water quenching, maintaining a large fraction of the unstable β-Ti phase, which pre-formed when heated up to 850 °C for the recrystallization process. A closer look at the β-Ti phase revealed that there was no Ti_2_Ni precipitate. The aging at 600 °C allowed the reprecipitation of the Ti_2_Ni to occur, which was only observed in the β-Ti phase, accompanied by the shrinkage of the β-Ti phase. Thermal energy can trigger the transformation of the unstable β-Ti phase into the stable α-Ti phase and Ti_2_Ni precipitate by expelling β stabilizing elements. The reprecipitated Ti_2_Ni in the β-Ti phase became separated and refined and was controlled by adjusting the aging temperature, which needed to be high for nucleation but not too high so as to suppress the growth of the precipitate.

**Figure 3:**
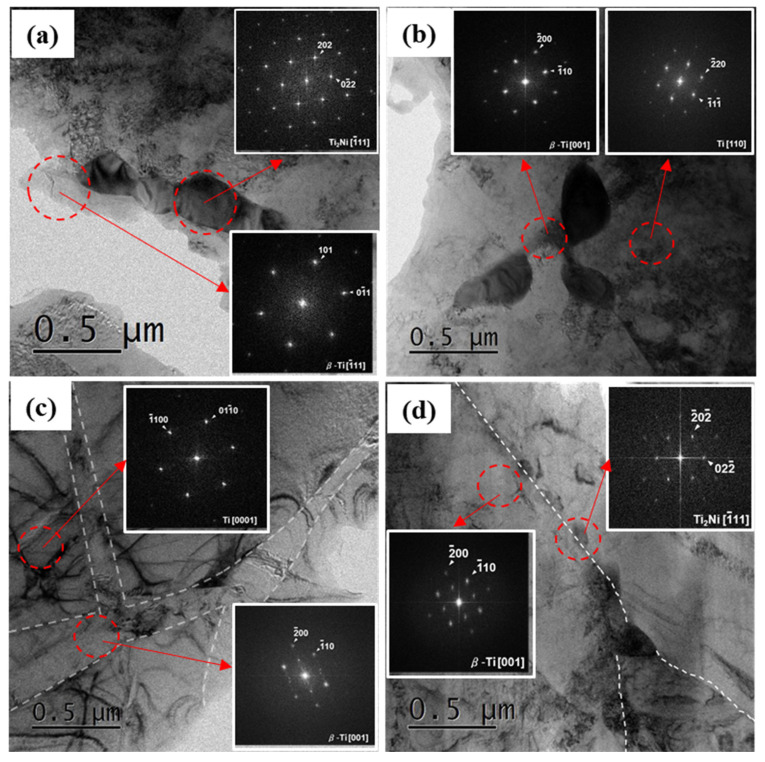
TEM images showing morphologies of each phase in (**a**) Furnace-cooled sample, (**b**) Gas-quenched sample, (**c**) Water-quenched sample and (**d**) Water-quenched + aged sample. The insets in each image are SAD patterns corresponding to each phase.

A TEM analysis was conducted to further investigate the second phase area. Figure 3 shows the microstructure of the second phase area, including the selected area diffraction (SAD) patterns corresponding to each phase and the compositional results of each phase, which are listed in Table 1.

Except for size, the furnace cooling and gas quenching cases showed a similar microstructure, including the composition of each phase. The Ti_2_Ni precipitate contained about 65 wt% Ti and 35 wt% Ni, including a minor amount of Ru, as low as 0.2 wt%. Most Ru existed in the β-Ti phase adjacent to the Ti_2_Ni precipitate. The α-Ti phase was revealed to be pure Ti, indicating that the alloying elements were consumed only for the β-Ti phase and Ti_2_Ni formation. The cooling rate of water quenching seemed to be too fast to cause phase transformation; therefore, the pre-formed β-Ti phase at recrystallization temperature, which contained all added Ru and Ni, was maintained after water quenching. As all β stabilizing elements were used for the β-Ti phase at this moment, its quantity could be maximized, as shown in Figure 2. Once phase transformation, such as precipitation and β to α transformation, occurred during the aging process at 600 °C, Ni and Ru elements were expelled from the β-Ti phase and redistributed, leading to the formation of Ti_2_Ni as well as the partial transformation of the β-Ti phase into the α-Ti phase. These microstructural changes had an impact on the composition of each phase. The Ti_2_Ni precipitate formed after aging at 600 °C with a lower Ni content than the others. Ni in the β-Ti phase after water quenching was fully used to the Ti_2_Ni precipitate during aging, while Ru in the β-Ti phase was consumed partially during aging, resulting in 4.4 wt% Ru in the β-Ti phase. However, this Ru content seemed to be fairly low compared with those of furnace cooling and gas quenching (~18 wt%), which was ascribed to the larger quantity of the β-Ti phase after aging.

### 3.2. Corrosion Properties

**Figure 4:**
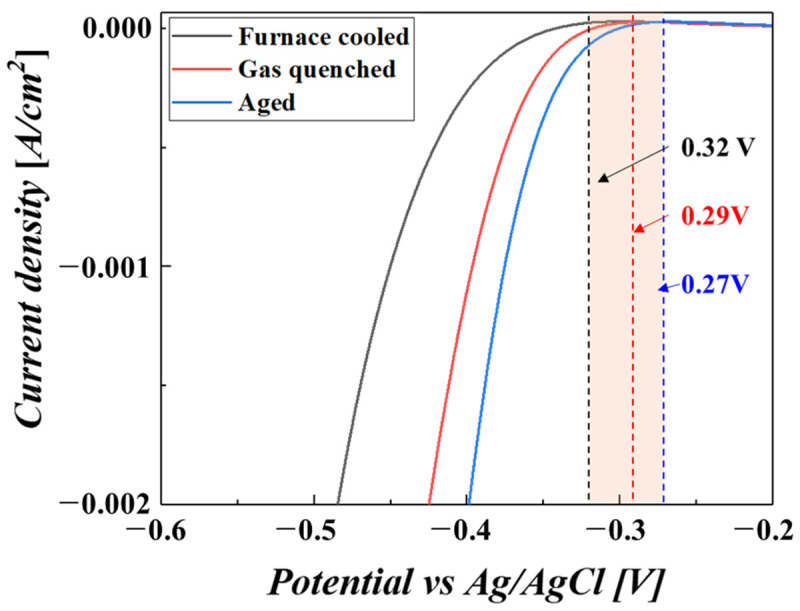
Linear sweep voltammetry results showing onset potentials for the hydrogen evolution reaction of the samples treated with different cooling rates.

As a main mechanism for improving corrosion resistance, the cathodic modification effect could be greatly influenced by the Ti_2_Ni precipitate in the Gr13 titanium alloy. A noble shift in the cathodic reaction could be strengthened by microstructural changes, such as precipitate refinement. Figure 4 shows the onset potential for HER. An improvement in the cathodic modification effect was related to reduced overpotential for HER [29,30,31].

As shown in Figure 2, the water-quenched sample combined with the aging process made the precipitate refined and separated, which is a favorable aspect for HER due to the increased surface area of the Ti_2_Ni precipitate. As a catalyst, the larger surface area of the precipitate provided more activity for HER. Therefore, the gradual refinement of the precipitate while increasing the cooling rate made the onset potential reduce sequentially from 0.32 V, 0.29 V and finally to 0.27 V.

**Figure 5:**
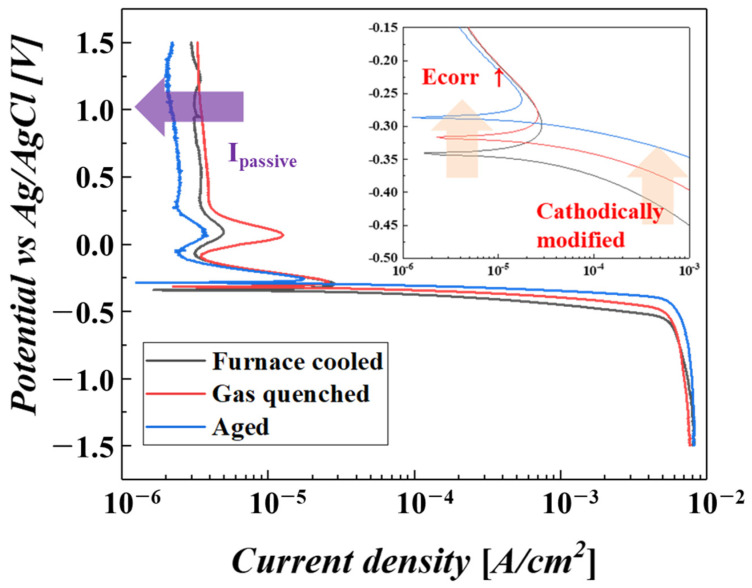
Polarization curves of the samples treated with different cooling rates. The inset is the enlarged part of the corrosion potential region, where E_corr_ is increased due to the cathodic modification effect.

**Figure 6:**
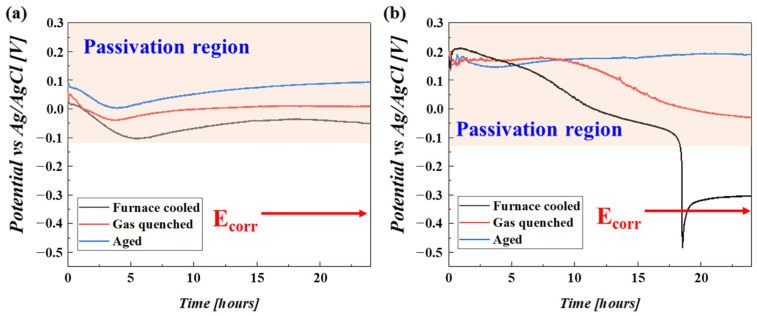
OCP variations in the samples immersed in (**a**) 10 wt% sulfuric acid solution and (**b**) 30 wt% sulfuric acid solution.

Figure 5 shows the polarization curves of three samples. Similar to the results of onset potential, the corrosion potential (E_corr_) was also cathodically shifted by increasing the cooling rate as a result of the cathodic modification effect. The inset in Figure 5 clarifies the evolutions of the cathodic modification effect with the cooling rate. The cathodic reaction increased with the heightened cooling rate due to reduced overpotential and, thus, led to an increase in corrosion potential [32,33,34]. This signifies that a higher cooling rate could provide more chances for passivation, which is ascribed to the catalytic effect of the precipitate. Also, the higher tendency for passivation results in a decrease in the passive current density (I_passive_). As mentioned in the introduction, cathodic modification was used for passivation materials, as the resultant effect increased its tendency to be passivated. The added elements, such as Ru and Ni, formed the Ti_2_Ni_(1−x)_Ru_x_ precipitate, and its contribution to HER induced a noble shift in the cathodic reaction, which could improve passivation stability and, thus, result in a decrease in I_passive_. The improved passivation stability was confirmed by an open circuit potential (OCP) test, as shown in Figure 6.

For the immersion in 10 wt% H_2_O_4_ solution, the OCP of the three samples were all maintained in the passivation region with immersion time. Only a slight difference in OCP among the samples was observed, which could result from the different cathodic modification effects. However, when the samples were placed in a 30 wt% H_2_SO_4_ solution, OCP behavior changed. The furnace-cooled sample showed an abrupt decrease in OCP because of a loss in passivation and was finally placed in the corroded state. The gas-quenched sample showed a gradual decrease in OCP instead of an abrupt decrease in OCP, meaning that passivation deteriorated [35,36,37,38,39,40,41]. While the OCP of the aged sample after water quenching remained almost constant with immersion time, it indicated that only by manipulating the cathodic modification effect could the passivation ability be improved, even in the case of the highly concentrated sulfuric acid solution.

Improved corrosion resistance with the cathodic modification effect was evaluated in terms of the corrosion rate based on weight loss. The calculated corrosion rate from weight loss is listed in Table 2.

The furnace-cooled sample displayed the highest corrosion rate, followed by the gas-quenched sample and, finally, the aged sample, whose corrosion rate was 10 times lower than that of the furnace-cooled sample. This remarkable increase in the corrosion rate of the aged sample could be related to the preservation of passivity during the immersion in 30 wt% sulfuric acid solution. Thus, it can be said that an improved passivation ability through the manipulation of the cathodic modification could be an effective method for further enhancing the corrosion resistance.

## 4. Conclusions

The cathodic modification effect could be further strengthened by only controlling the microstructure of the Ti_2_Ni precipitate without any compositional changes. Gr13 consisted mainly of the α-Ti phase, where the Ti_2_Ni precipitate acted as a catalyst for HER, forming wither with or in the β-Ti phase and around the grain boundary. Due to the slow cooling rate as low as 3.7 °C/min, a coarse Ti_2_Ni precipitate was formed, along with the grain boundary for the furnace-cooled sample. Even gas quenching increased the cooling rate as high as 160.9 °C/min, which also resulted in a coarse precipitate but made the precipitate slightly narrower than that of the furnace-cooled sample. However, there was no precipitation formation in water quenching due to the fast-cooling rate. Only the β-Ti phase was observed as a second phase, which was pre-formed when heated up to 850 °C for the recrystallization process. Aging at 600 °C resulted in the reprecipitation of Ti_2_Ni, though, during this moment, its microstructure differed: it became more refined and separate. In terms of the reactivity for HER, the characteristics of the precipitate encouraged HER, resulting in a reduction in the onset potential of HER and, thus, strengthened the cathodic modification effect. This improvement, in turn, reduced the corrosion rate, and the aged samples showed a 10 times lower corrosion rate than that of the furnace-cooled sample because of passivation survival. As a result, the enhancement of the passivation ability enabled the passivation state to be maintained for 24 h of immersion in 30 wt% sulfuric acid solution, resulting in the lowest corrosion rate.

## Figures and Tables

**Table 1:** Compositional analysis on each phase of the sample.

	Furnace Cooling	Gas Quenching	Water Quenching	Water Quenching + Aging
Ti_2_Ni	Ti 65.6 wt% Ni 34.2 wt% Ru 0.2 wt%	Ti 64.7 wt% Ni 35.3 wt%	-	Ti 70.1 wt% Ni 29.9 wt%
β-Ti	Ti 78.5 wt% Ni 3.0 wt% Ru 18.5 wt%	Ti 82.8 wt% Ru 17.2 wt%	Ti 86.0 wt% Ni 8.6 wt% Ru 5.4 wt%	Ti 95.6 wt% Ru 4.4 wt%
α-Ti	Ti 100 wt%	Ti 100 wt%	Ti 100 wt%	Ti 100 wt%

**Table 2:** Calculated corrosion rate based on weight loss.

Sample		Corrosion Rate [mm/yr]	Average Corrosion Rate [mm/yr]	Standard Deviation
Furnace cooling	1	3.83	4.42	0.54
	2	5.14		
	3	4.29		
Gas quenching	1	2.04	1.82	0.24
	2	1.93		
	3	1.49		
Water quenching + aging	1	0.41	0.41	0.13
	2	0.25		
	3	0.57		

## Data Availability

The data presented in this study are available on request from the corresponding author.
